# ‘Physical well‐being is our top priority’: Healthcare professionals' challenges in supporting psychosocial well‐being in stroke services

**DOI:** 10.1111/hex.14016

**Published:** 2024-03-12

**Authors:** Felicity A. S. Bright, Claire Ibell‐Roberts, Katie Featherstone, Nada Signal, Bobbie‐Jo Wilson, Aileen Collier, Vivian Fu

**Affiliations:** ^1^ Centre for Person Centred Research Auckland University of Technology Auckland New Zealand; ^2^ Geller Institute of Ageing and Memory University of West London London UK; ^3^ Department of Physiotherapy Auckland University of Technology Auckland New Zealand; ^4^ College of Nursing and Health Sciences Flinders University Adelaide South Australia Australia; ^5^ Medical Research Institute of New Zealand Wellington New Zealand; ^6^ Department of Clinical Neurosciences University of Calgary Calgary Alberta Canada

**Keywords:** healthcare practitioner, professional practice, psychosocial well‐being, qualitative, stroke

## Abstract

**Background:**

Following stroke, a sense of well‐being is critical for quality of life. However, people living with stroke, and health professionals, suggest that well‐being is not sufficiently addressed within stroke services, contributing to persistent unmet needs. Knowing that systems and structures shape clinical practice, this study sought to understand how health professionals address well‐being, and to examine how the practice context influences care practice.

**Methods:**

Underpinned by Interpretive Description methodology, we interviewed 28 health professionals across multiple disciplines working in stroke services (acute and rehabilitation) throughout New Zealand. Data were analysed using applied tension analysis.

**Results:**

Health professionals are managing multiple lines of work in stroke care: biomedical work of investigation, intervention and prevention; clinical work of assessment, monitoring and treatment; and moving people through service. While participants reported working to support well‐being, this could be deprioritised amidst the time‐oriented pressures of the other lines of work that were privileged within services, rendering it unsupported and invisible.

**Conclusion:**

Stroke care is shaped by biomedical and organisational imperatives that privilege physical recovery and patient throughput. Health professionals are not provided with the knowledge, skills, time or culture of care that enable them to privilege well‐being within their work. This has implications for the well‐being of people with stroke, and the well‐being of health professionals. In making these discourses and culture visible, and tracing how these impact on clinical practice, we hope to provide insight into why well‐being work remains other to the ‘core’ work of stroke, and what needs to be considered if stroke services are to better support people's well‐being.

**Patient or Public Contributions:**

People with stroke, family members and people who provide support to people with stroke, and health professionals set priorities for this research. They advised on study conduct and have provided feedback on wider findings from the research.

## 1 BACKGROUND

The number of people living with stroke in Aotearoa New Zealand (referred to as ‘Aotearoa’ in this paper) is projected to grow as the population increases and ages.^1^ Stroke affects physical, cognitive and communicative function^2^ and people's psychological, social and emotional well‐being.^3^, ^4^ These impacts can extend beyond the person to their wider whānau (a cultural construct that includes family and extends to those within a person's wider network who they identify as important to them).^5^ Despite this, stroke care predominantly focuses on acute and subacute care, prioritising recovery of impairments, maximising physical function and preventing future strokes.^6^ The last 50 years have witnessed significant developments in medical management of stroke,^7^ and in Aotearoa, investment in improving early stroke care, particularly through medical interventions.^8^ Amidst the focus on early management, and physical recovery from stroke, the wider impacts on well‐being and quality of life can be overlooked.

Well‐being is increasingly recognised as important after stroke. While well‐being is multifaceted,^4^ for this paper, it refers to people's psychological, social and emotional well‐being. People can experience significant changes in identity,^9^ hope,^10^ friendships,^11^ mood^12^ and other aspects of well‐being. People with stroke report that services fail to address their well‐being needs,^3^ which can exacerbate the impact of stroke.^4^, ^13^ Health professionals, including allied health clinicians, nurses and doctors, can play a critical role in supporting well‐being through interpersonal interactions;^14^, ^15^ however, interpersonal communication remains a primary form of ‘missed care’ (necessary care that is not provided).^16^ Previous research suggests that health professionals do not feel skilled to support well‐being,^17^ and can feel that focusing on well‐being in stroke care is in conflict with their disciplinary priorities,^18^ despite practice guidelines stating that ‐‘well‐being is everyone's business’.^19^ Even though health professionals express a desire to better support well‐being,^17^ and patients advocating for well‐being to be a priority in research and practice,^20^ it remains largely unaddressed within services.^21^

It may be valuable to understand how the practice context informs how health professionals address well‐being. The systems and structures that shape stroke care are known to strongly influence practice.^18^, ^22^ The prevailing emphasis on biomedical needs, physical impairments and function, and organisational priorities of discharge^22^ are shown to present challenges in enacting person‐centred care.^23^ Indeed, health professionals who have transitioned into roles *outside* medically dominated systems of care report a sense of freedom and flexibility to engage with people, to actively listen and engage in interactions that support well‐being.^24^, ^25^, ^26^ Rather than assuming that health professionals are solely responsible for not addressing well‐being, it is critical to examine how the contexts of care shape practice and how this impacts on how well‐being is addressed within stroke services.

### 1.1 Research aims

The aim is to understand how health professionals address well‐being of people with stroke, and to examine how the practice context of stroke services influences care practice.

## 2 METHOD

### 2.1 Study design

The study is an interview‐based study of health professionals' perspectives and experiences of addressing well‐being after stroke. It sits within a larger programme of research examining well‐being after stroke; within the wider research, we explore the perspectives of people with stroke, their families and national guidelines.

We utilise Interpretive Description,^27^ a qualitative methodology designed to provide insight into practice‐oriented issues and to generate findings that can be applied to and taken up in practice settings. Our approach has an Institutional Ethnography^28^ orientation, supporting a focus on understanding how clinical practice is managed and organised by larger ruling relations and how this shapes people's understandings of what they *should* do.^28^

### 2.2 Context of stroke care in Aotearoa

People with suspected stroke are admitted to the local Emergency department. After initial assessment, people may be managed locally or transferred to a tertiary hospital for specialist input. Large urban hospitals commonly have stroke‐specialist services, while nonurban care is provided by general medical teams.^29^ Care is provided by multidisciplinary teams, with medical, nursing and allied health services. Some have clinical nurse specialists (CNS) who play a role in care co‐ordination, often over the continuum of care. The minority of services have access to psychology. All hospitals offer some form of inpatient rehabilitation, used by around one‐third of stroke patients. Community rehabilitation services may be general or stroke‐specialist; 50% are time‐limited, with people able to access services for 6 weeks to 3 months.^29^ National quality indicators require transfer between services within specified time periods.^30^

### 2.3 Sites and participants

Ethical approval was obtained from the Auckland University of Technology Ethics Committee (AUTEC 21/223). Our research was centred in two District Health Boards (DHBs), with locality approval. These DHBs cover urban, regional and rural settings, and between them, offer specialist stroke, and general medical and rehabilitation services. They both serve high Māori populations, the Indigenous population of Aotearoa. However, to extend our understanding of the wider stroke context outside these two DHBs, we also recruited participants working in stroke care throughout Aotearoa through personal and professional networks, and snowball sampling of people suggested by initial participants.

Eligible participants were health professionals working in stroke care in Aotearoa. Purposive sampling sought a diverse mix of disciplines, roles, types of stroke service and professional experience. Potential participants were provided with brief information and invited to contact researchers to indicate interest in participating. All participants provided informed consent before their interview.

### 2.4 Data gathering

Data were gathered through semistructured individual interviews (*n* = 26) and joint interviews (n = 1, 2 participants), reflecting that colleagues wished to be interviewed together. An interview guide was developed (Appendix A). Participants provided brief demographic information. Participants in the two participating DHBs were invited to share local policies and guidelines.

Interviews ranged from 33 to 92 min (average = 59 min). Several participants emailed the researchers after the interview, adding reflections. These were included as data.

### 2.5 Data analysis

Analysis was iterative, starting with familiarisation.^31^ Analysis was led by F. B. and C. I.‐R. We wrote brief notes immediately following each interview, and then generated detailed reflective memos. This process suggested that health professionals experienced significant tensions when addressing well‐being. Following analysis meetings with the wider research team, we then deepened analysis using applied tension analysis.^32^ We identified tensions (i.e., points of strain, challenge of inconsistency), coding for *praxis tensions* identified by participants and *conceptual tensions* identified by the researchers, then examining the contexts that gave rise to those and the enacted responses (i.e., how people navigate these tensions). These were thematically grouped, and we examined how contexts constrained and/or enabled health professionals to attend to psychosocial well‐being.^32^ At this stage, we reviewed local and national policies and guidelines that people mentioned in interviews, to understand the practice context. Through this process, the notion of ‘work’ and how different elements of the ‘work’ of stroke care impacted on how people addressed well‐being became evident in the different themes. Regular analytic discussions were held with the research team, and all team members contributed to refining and writing the analysis presented here.

Research credibility was achieved through multiple strategies, grounded in our methodology.^27^ Gathering data across multiple contexts and participants, supporting analysis with raw data and situating the analysis within the wider context of stroke care support *representative credibility. Analytic logic* is demonstrated through detailed description of the research process. *Interpretive authority* arises from the robust analysis approach and team discussion, with researchers bringing expertise across health disciplines, and with expertise in stroke research, and organisational research.

## 3 RESULTS

Twenty‐eight health professionals participated in interviews in 2022 and 2023 (see Table 1). Participants spanned a range of disciplines and continuum of care. Five held joint clinical and leadership roles (e.g., Charge Nurse Manager, Allied Health Lead). Participants had a median 12.5 years of clinical experience.

**Table 1:** Participant characteristics.

Gender (self‐identified)	
Male	3
Female	25
Ethnicity	
New Zealand European	19
Māori	2
Indian	1
Other	6
Median postqualifying experience (range)	12.5 (2–41)
Area of practice (many worked across multiple areas)	
Hyperacute/acute	15
Inpatient rehabilitation	17
Community rehabilitation	15
Stroke service type	
District Health Board	25
Nongovernmental organisation (e.g., charity, not‐for‐profit)	3
Role and discipline (reflecting that people may have roles outside their disciplinary role)	
Physiotherapist	5
Occupational therapist	5
Speech‐language therapist	3
Social worker	3
Psychologist	1
Nurse	8
Doctor	2
Clinical leadership or management role	5
Other (e.g., service co‐ordinator)	2
Gender (self‐identified)	
Male	3
Female	25
Ethnicity	
New Zealand European	19
Māori	2
Indian	1
Other	6
Median post‐qualifying experience (range)	12.5 (2–41)
Area of practice (many worked across multiple areas)	
Hyperacute/acute	15
Inpatient rehabilitation	17
Community rehabilitation	15
Role and discipline (reflecting that people may have roles outside their disciplinary role)	
Physiotherapist	5
Occupational therapist	5
Speech‐language therapist	3
Social worker	3
Psychologist	1
Nurse	8
Doctor	2
Clinical leadership or management role	5
Other (e.g., service co‐ordinator)	2

Stroke services in Aotearoa prioritised three primary lines of work, which then shaped what work was prioritised. These were as follows:

1. The biomedical work of investigation, intervention and prevention.
2. The clinical work of assessing, monitoring and treating impairments and function.
3. The work of moving people through services.

The analysis also revealed a fourth line of work: the work of supporting well‐being. While recognised as important, the work of supporting well‐being could be deprioritised or lost amidst the lines of work that are privileged within stroke services.

### 3.1 The biomedical work of investigation, intervention and prevention

Stroke is a medical emergency. From the time a potential stroke is recognised, a series of events are triggered. Preadmission communication results in a ‘code stroke’ being called:

[The stroke team] run to the stroke call, attend the patient, assess everything very quickly. Get the scan done and decide whether to give reperfusion therapy. And if we decide to do so, then would we go ahead with just thrombolysis or have to send them to [tertiary centre] to have clot retrieval? (CNS)

This quote conveys the urgency of this care, reflecting the limited time window for reperfusion therapies. This pace of care is fast for the patient and whānau; it is also fast for the team.

Early stroke care focuses on ‘keeping [people] alive and improving their outcomes and getting them medically stable’ (CNS), shaping care priorities:

[I'm] aiming for diagnosis, investigation and treatment at the same time. So I confirm diagnosis and then the rest of investigation will be try to pick out why they have [had a] stroke, so we could try to prevent it again. And that investigation part [goes] alongside the treatment. (Doctor)

These priorities reflect local and national guidelines. Guidelines from one service expected ‘best practice management’ in the hours after stroke centred on pharmacological and surgical treatment; expected care in the *days* after stroke focused on physiological support, prevention and management of complications and secondary prevention. This structured the work of nursing and allied health, who described their early priorities as preventing complications and planning future treatments:

You also need to do a full neuro screen to see, how do we need to make sure we've set them up so that we're protecting them … and so that the nurses know the best way to mobilise and support them for their cares. And the family know how to support their upper limb if it's swollen or how to prevent sublux. (Physiotherapist)

The quotes in this section show how practice descriptions focus on clinical, technical aspects of stroke care. Despite a potentially life‐changing and often distressing event for patients and whānau, the focus is on the physical aspects of care. While time‐limited, the resulting line of work reflects and effects a technical, biomedical orientation to care^33^ that is taken up and continued as people progress through stroke services.

### 3.2 The clinical work of assessing, monitoring and treating impairments and function

Participants reported that the orientation towards physical care continues throughout stroke care, with health professionals prioritising assessment and treatment of people's physical, cognitive and communicative impairments and function:

I do feel we then move to impairment‐based stuff quite quickly. I think we're aware that there isn't much out there so it's almost like a rush to give them the best chance and by that chance we're prioritising impairment‐based. (Speech‐language therapist)

What constitutes ‘best chance’ appears to reflect a biomedical frame of resolution of impairments. Prioritising physical recovery was also evident in participants' descriptions of how they managed their therapeutic time. Mindful that services were short term, with little follow‐up available, participants described protecting therapeutic times to ensure that they had time to work on areas that they viewed as directly related to their disciplinary expertise:

Time's precious so you wanna be making the most of your hour or so you've got with them rather than spending 20 minutes grieving or talking. you can still talk them through things but if they're having huge emotions, struggles with their relationship … or how this is gonna affect their ability to go home, [I want] someone else that can kind of take some of that. (Physiotherapist)

However, this quote reflects an assumption that physical and emotional needs can be separated. Some suggested that once ‘all the practicalities’ were addressed (such as toileting, mobility, arm function), *then* well‐being could be addressed, a linear view of what aspects should be addressed at what time points:

On the ward, you're very much about survival and what do you need in order to get home and how can we support you in order to get home …. [Then] okay, now we're home, we know you are safe enough to survive the day, what do you want to do now? (Occupational therapist)

The dualist separation of physical and psychosocial well‐being can have significant, unintended consequences that some participants recognised:

Physical wellbeing is our top priority and is generally done really really well, but I would say all other aspects, emotional, psychosocial, very poorly addressed, if at all. If I'm brutally honest … It doesn't matter how you are actually physically after a stroke, if you're traumatised by it and you're depressed, clinically depressed, then that's just going to be so detrimental to your life regardless of your physical state. (CNS)

One site had patients who were described as ‘long stayers’—those not seen to be benefitting from physical rehabilitation but were awaiting placement in residential care—and were recognised to have well‐being needs. Nevertheless, the priority given to physically oriented care meant that although acknowledged, these aspects of well‐being were not expressly addressed in the ward setting:

Basically they just live on the ward which [is] really hard for them … as a therapist you kind of tap out because they're independently mobile and … there's so much you could provide them but when you're weighing up what your priorities are, they kind of just get left loitering around a ward … it's really sad in that respect … you presume they would definitely need some sort of emotional support or social support. (Physiotherapist)

While the impact of separating physical well‐being and psychosocial well‐being was recognised by many participants, they reported little institutional support to address well‐being. They indicated that attending to physical needs and the routinised work of care took much of the clinical time that they had available, given high numbers of patients, and high dependency of patients. In this context of busyness, health professionals described seeking to do their best, but knew that they offered different care to what might be seen as ideal:

[Nurses are] doing it so fast they're also trying to support their patient as much as they can and they just, all they can remember is the task that they've done or haven't done, and how that makes them feel like they haven't provided the best care for the patient. (CNS)

The quote demonstrates how the sense of pressure that staff felt to address the immediate physical needs while knowing that there are other, significant unmet needs could take its toll.

### 3.3 The work of moving people through services

Moving patients through services in a timely manner is prioritised and driven in part by national stroke quality indicators.^34^ Hospital‐based services face significant occupancy demands, with staff being directed to prioritise patient movement, flow and discharge:

It's the perspective of an inpatient hospital we need to discharge people … the hospitals are 80, 90, 100 percent capacity. … We've got … to keep people moving. (Physiotherapist)

Combined with requirements for timely transfer between services, this focus on ‘moving people’ placed pressure on staff and what they prioritised. Participants expressed their frustration at patients being discharged without full assessments and supports:

Forty‐one, single, working, with some right‐hand dexterity problems and a right facial droop and some dysarthria. And I didn't get to see her … I rang up‐ [the medical team] and said, ‘Hang on, don't discharge her, she needs to see OT. She could do with seeing social work.’ She never got to see social work, ‘cause they just discharged her. (CNS)

Discharge criterion reflected biomedical frames of medical stability, physical safety and function at home (with support); these also drove treatment.

This pressure for throughput was recognised to be important in community services also:

That allows us to kind of be that reactive service in which we can see people within seven days and we can get out to see people urgently and provide the ESD [early supported discharge] service. I guess you've gotta weigh up if you keep people on for a long time, then you can't really provide that ESD, early supported discharge. (Physiotherapist)

Health professionals sought to manage their responsibilities to the people they already see *and* to *future* patients through a continued orientation to moving people through services.

However, in the drive to discharge, participants suggested that the impacts of stroke, ‘the things that we think are minor, and are minor in a clinical sense, [but] really aren't minor to that person’ (CNS), could be left unseen. In the context of biomedical orientations to care, and pressure to discharge, it was challenging for staff to prioritise well‐being: ‘I think there's a place for [addressing well‐being]. I just don't think there's the resources for it’ (Physiotherapist).

### 3.4 The unsupported and often invisible work of supporting well‐being

Amidst the work of stroke care was the work to support well‐being. This work was seemingly invisible, enacted through small interactions, but absent in policies and procedures, assessments, patient records and key performance indicators.

Participants described attending to well‐being in three ways. First, they addressed well‐being issues in the moment, acknowledging emotions, considering what they might be able to do at that time to support people. In Emergency, this oriented to providing information, reassurance and comfort: ‘[if there are other senior clinicians present, then] my job is to be with that patient and hold their hand and tell them it's okay’ (CNS). In later stages in care, this relied on the health professional picking up the ‘flags’ and ‘clues’ that things were not alright. Well‐being was supported by small acknowledgements of these signs and talking about these with the person:

I just caught snippets here and there as I was talking to them. Little flags. She'd sent out some flags … I find people when they want help around psychosocial issues, will not be direct with you, because through embarrassment and the stigma that still exists. I find they will send out little clues to you. And you can only pick up on those clues by actually having the time to sit and talk to them and making them feel relaxed. (CNS)

While this form of well‐being work was universally seen as important, it was not without tension:

People feel pressured to get their session done and move on or do something that day in relation to your role and so … no‐one takes hold of that emotional well‐being side of things and that just pulls away because the patient's doing physio, SLT and OT, but not other things. (Speech‐language therapist)

This tension between so‐called disciplinary work and well‐being work gave rise to the other approaches to supporting well‐being. Second, some participants put confines around what they would support, such as ‘listening and reassurance, and providing education on specific rehab whether its interventions or tools or services … but in terms of the grief and loss… that's not me, that's beyond me’ (Physiotherapist). People recognised the importance of well‐being but appeared to struggle to know how to balance the different demands, in the context of the time available and their own knowledge and skill. As such, the third approach was to look to others to provide greater support, particularly to social work, or in community, referring to the general practitioner: ‘My solution is to go to your GP and get [depression medication]. … I just have to send them back to their GP’ (CNS). When asked if health professionals or stroke services did anything different for Māori, few participants offered any specific suggestions of working differently to address well‐being.

The work to support well‐being and the *pace* at which these health professionals needed to work to support well‐being were at odds with the pace needed to complete their other work. The notion of ‘busyness’ was repeatedly raised, such as ‘The social workers are busy with the discharge planning’ (Physiotherapist) and ‘the nursing staff are so busy’ (Nurse Manager). This conveyed a sense of time‐pressured staff working with only so much time to give. Participants were aware that busyness could be problematic: ‘People are far more attuned to how important [well‐being work] is. it's not down time—it's essential time to build in. But as a therapist, you're focused on getting the maximum out of the time that you have’ (Physiotherapist). Nurses described how times of relative slowness on the wards created space for them to support well‐being—the evenings, the weekends—the times when the rush of other staff and their work dissipated.

While many aspects of stroke care are defined and detailed in protocols, guidelines and forms, the work of well‐being is missing and risks being delegitimised. Many participants struggled to describe how they addressed well‐being, suggesting that it was hard to articulate and show what they do to themselves and to others, rendering this work invisible and untraceable. One CNS said:

I think a lot of staff do try [support well‐being] in their practice, but they [aren't] necessarily able to label what they're doing or what it means to the patient …. they know it's important but they just don't know how to like label it.

The lack of language perpetuates the invisibility of well‐being in services but this also reflects that well‐being was not learned during their training. As a result, well‐being work was often constructed as *other to* people's core disciplinary work, meaning that they did not feel confident that they were providing the most skilled and appropriate support: ‘I guess you would say we muddle through … we do our best but could we be doing better? Probably.’ (Physiotherapist).

The emotional impact of knowing patients needed support, and not being able to provide it, or not having anywhere to refer to, weighed heavily on some participants. Several nurses described a deep worry that they had for patients who they knew were struggling or were likely to struggle. Months later, one commented how a patient was ‘still on [her] mind’, knowing that the patient may have significant psychosocial impacts. Another nurse, working in a team with minimal follow‐up after discharge, said:

It is hard to just say, ‘Oh well that's, see you later. This is your life now.’ And it is really taking a toll, ‘cause I do care about them, as much as I probably shouldn't, but I do care about my patients, I make sure that they're okay. And I would love to have a solution’. (CNS)

Beyond referring to General Practitioners, who were not seen to have solutions other than medication, people felt that there was little they could do.

However, several participants described a different approach to considering and supporting well‐being. They considered that providing well‐being support and addressing people's wider needs beyond impairments and function was a core component of their role. One Māori social worker described her approach to practice: ‘It's not always just medical and psychological, it's about whānau. it's about feeling connected, feeling loved, feeling accepted, even though my body's like this’. This framing opened up different possibilities for care, and different ways for her to work, viewing herself as a facilitator, opening up space for people to experience well‐being. Holding space for well‐being and resisting dominant messages about stroke care appeared to be enabled by a strong philosophy of practice and was supported by strong leadership, with one social worker saying: ‘if you get someone to lead it, and teach it, then it grows’. Taking a holistic approach to well‐being challenges disciplinary, physically based care that centres the health professional, their skills, their scope and remit. It prompts different ways of thinking and constructing stroke care, and resists the rapid pace and push towards discharge.

## 4 DISCUSSION

This study provides new insights into how the context and culture of stroke care shape clinical practice. Our findings demonstrate that this results in health professionals privileging care relating to physical recovery, early disciplinary‐based intervention and movement through services. Well‐being work took a particular form: supporting people's emotions and considering mood‐related issues. In many ways, it was predominantly reactive rather than proactive, and not attending to the wider constructs of well‐being. Work recognised as essential for well‐being such as culturally informed practices,^35^ engagement and support of whānau^35^, ^36^ and building people's psychosocial resources to support their longer term recovery^37^ were rarely privileged within services in our research. While rehabilitation *could* be a time when people are supported to consider long‐term well‐being, and find meaning in their poststroke world,^38^ instead, it appeared to be assumed, or perhaps hoped, that well‐being would naturally develop as physical recovery occurred. While physical recovery has been identified as a key priority by people with stroke,^39^, ^40^ it is clear that it is not the *only* priority,^40^ and there is persistent evidence that well‐being remains as an unmet need after stroke.^3^, ^41^

Time was seen as a limited resource, something that staff needed to carefully manage to ensure that they contributed to organisational goals of patient throughput,^42^, ^43^ productivity and efficiency.^44^ This created urgency and busyness for health professionals, although this is not reflective of patients' experience, for whom boredom and loneliness are common.^45^, ^46^ While participants in our study were aware that psychosocial lines of work were ‘left undone,’^16^ the focus on patient throughput had a strong influence over what processes and practices were legitimised and prioritised in care.^47^ Lines of work prioritised in stroke care within this study reflect ‘the increasing technical, biomedical objectification of stroke unit provision’.^33^ Discourses of neuroplasticity, compounded by time being constructed as limited, saw many health professionals prioritise intensive intervention in the first months after stroke, seeking to maximise the ‘window’ for recovery.^48^ This view of recovery commonly focused on particular functions—body structures and functions, and activities of daily living.^49^ Viewing recovery in this way may perpetuate mind–body dualism^50^ and may be a key factor in why people with stroke consider that their well‐being is not well addressed.^21^ This reflects linear views of what aspects of care and recovery should be prioritised when, and perhaps reflects particular understandings of what aspects of care, and what functions in life, are considered in and out of scope for services. This may also suggest an assumption that physical improvements will improve, and are central to, wider well‐being.

Stroke care is deeply embedded within Western worldviews.^51^ The resulting lines of work perpetuate monocultural care that fails to recognise the experiences of Māori,^36^, ^52^ does not acknowledge or uphold whānau contexts that extend beyond individualistic notions^36^ nor does it reflect holistic views of well‐being important for Māori after stroke.^4^ The fact that few participants indicated how they worked differently with Māori is indicative of a dominant monocultural approach to care. Moreover, the pace of care can be at odds with allowing time and space for meaningful connections^53^ between whānau and the clinical team. While some health professionals in this study worked to centre whānau needs and aspirations, this required pushing back against dominant cultural and clinical perspectives. This study provides insight into why many whānau are required to leave much of themselves ‘at the door’^54^, ^55^ to engage with stroke services, a necessity at odds with an approach to care that fosters well‐being.

Our research shows that health professionals are aware that people need support for well‐being, and yet often feel unable to provide or prioritise it. The reluctance to ‘[open] up a can of worms’^56^ emanated from fear that patients would raise issues that they feel ill‐equipped or poorly resourced to address.^57^ This reflects what is taught within student education^58^ and what is valued in wider professional paradigms.^59^, ^60^ These shape what is viewed as ‘real work’,^47^ what aspects of a patient's experience capture people's attention^59^ and crucially, what areas of practice they feel confident and knowledgeable to address. When health professionals feel that they do not have the skills and knowledge to support people's well‐being, it contributes to discomfort, guilt,^18^, ^61^ distress^33^ and inadequacy.^61^, ^62^ The awareness of ‘missed care’^16^ contributed to feelings of frustration, powerlessness and resignation in our participants; over time, this might contribute to disengagement^62^ or dehumanisation^63^ and potentially, moral injury.^64^ This may be exacerbated by the coronavirus disease pandemic, which has placed significant pressures on healthcare systems, staff and on communities. Within the current culture of care, health professionals may manage their emotional response by focusing on the areas where they feel they have knowledge, skills and competence, doing their best in the situation. Our work suggests that addressing health professionals' experiences is imperative to enabling holistic support for people with stroke as care that supports the *whole* person can only take place within systems that value and uphold the well‐being and personhood of *all*—patients, family *and* health professionals.^21^, ^23^, ^65^, ^66^

The prevailing culture of stroke care makes it challenging to support well‐being,^17^ and it is unsurprising that well‐being is not prioritised. However, we posit that the status quo is deeply problematic for health professionals *and* for people with stroke. It should not be accepted as normal that people with stroke navigate the reconstruction of self and life on their own, with little support,^67^ feeling isolated and abandoned by stroke services.^68^ We contend that it should not be seen as inevitable that people have significant reductions in friendships,^11^ and quality of life,^69^ or that 20% of people experience anxiety after stroke^70^ and 30% experience depression^71^—rates doubled if someone has aphasia.^72^ This raises pressing questions about the overarching objectives and priorities of current stroke service approaches. We ask whose needs are being served by current approaches to stroke service delivery and whose needs are not being met? It is not just feasible, but imperative, for health professionals to embed well‐being work into their routine practice. Some local changes may be able to be readily implemented, such as development of pathways for psychosocial support, staff training in supporting well‐being, inclusion of well‐being in core documentation and work processes within stroke services^19^ and creation of physical environments that support well‐being.^73^ However, practice changes require systematic support and resourcing, and fundamentally, changes to the culture of stroke care and what is considered a good outcome. This might include changes in key performance indicators at local and national levels, changes to undergraduate education to build knowledge and skills from early in one's professional development and more comprehensive attention to wider constructs of well‐being within stroke guidelines. These are just some suggestions, and we recommend that solutions are best developed through collaboration with health professionals, people with stroke and whānau, researchers and service leaders and policy‐makers. People with stroke live with the impacts of a stroke system that is designed around biomedical, short‐term care for a life‐long condition and deserve services that support them to thrive, not just survive.

## 5 LIMITATIONS

Our findings relate to health professionals' experiences within health systems in Aotearoa, which may impact transferability of findings. While we spoke to a range of health disciplines, the majority were allied health or nursing, and the nurses and doctors who participated in this research were all in specialist roles. It is likely that ward nurses and junior doctors may offer different perspectives. Our participants were predominantly tauiwi (non‐Māori); engaging with more Māori health professionals may have offered different insights into practice.

We do not wish to suggest that stroke services are the only form of support for people's well‐being. Peer support from others with stroke and whānau support from people's wider connections are invaluable in supporting well‐being.^4^ However, we do argue that stroke services have a critical role to play in supporting well‐being. We also acknowledge that in reading this work, health professionals may feel that the research team is judging their practice. Rather, our intent has been to show how practice is situated, shaped by the wider discourses and cultures of care that health professionals train and work within.

## 6 CONCLUSION

This study underscores that despite significant advances in stroke care, there is a significant disconnect between the prevailing biomedical focus of stroke care and the pressing need for holistic support for people's well‐being. Engaging with health professionals' experiences has revealed how service provision is shaped by entrenched biomedical paradigms and organisational imperatives that privilege physical recovery and patient throughput. Well‐being is invisible in this context. Health professionals are not equipped with the knowledge, skills, time or culture of care that enables them to privilege well‐being within their work; this has implications for the well‐being of people with stroke and the well‐being of health professionals. In exploring the culture and discourses within which stroke care occurs and tracing how these impact on clinical practice, we hope to challenge the status quo, and, ultimately, contribute to changes in culture and practice that better support the well‐being of all.

## AUTHOR CONTRIBUTIONS

**Felicity Bright**: Conceptualisation; funding acquisition; methodology; data collection; formal analysis; writing; validation. **Claire Ibell‐Roberts**: Data curation; formal analysis; project administration; writing—review and editing; investigation; validation. **Katie Featherstone**: Conceptualisation; funding acquisition; formal analysis; writing—review and editing; methodology; validation. **Nada Signal**: Funding acquisition; writing—review and editing; validation. **Bobbie‐Jo Wilson**: Methodology; validation; conceptualisation. **Aileen Collier**: Funding acquisition; writing—review and editing; validation. **Vivian Fu:** Funding acquisition; validation; writing—review and editing.

## CONFLICT OF INTEREST STATEMENT

The authors declare no conflict of interest.

## ETHICS STATEMENT

Ethical approval was obtained from the Auckland University of Technology Ethics Committee (AUTEC 21/223).

## Data Availability

Research data are not shared as per Ethics approval and participant consent.

## APPENDIX A INTERVIEW GUIDE

1. What do you think people need in order to flourish after a stroke?
2. What do you do in your role to support people's psychosocial well‐being?
3. Are you able to think of an example of a session with a patient and whānau during which well‐being issues have come up (whether you noticed issues, or patient or whānau raised them)—could you talk me through the session? If I was shadowing you, what would I see and hear you doing?
4. Do you find that people raise well‐being issues? If so, what is it that makes it possible for people to do so? If not, what do you think might be behind that?
5. We've been looking at literature which talks to how well‐being is not just affected by the health condition, but also by people's social context, inequity, and marginalisation. Is this something you see with stroke patients? Could you tell us more? How do you support well‐being after stroke when someone is coming in with significant challenges and already experiencing significant threats to well‐being?
6. Do you do anything differently to support Māori whānau well‐being?
7. When you think about a patient's episode of care in your service:
  a. Are there points where you feel your service does a really great job of addressing well‐being?
  b. Are there any missed opportunities that you notice?
8. If you imagine that addressing psychosocial well‐being needs was the primary focus of your service, and your service did a really great job of it:
  a. What would that look like?
  b. If you were going to make that change, what would need to change and how would you do it?
